# Thyroid-Like Follicular Carcinoma of the Kidney in a Patient with Skull and Meningeal Metastasis: A Unique Case Report and Review of the Literature

**DOI:** 10.1097/MD.0000000000003314

**Published:** 2016-04-18

**Authors:** Liang Dong, Jiayu Huang, Luke Huang, Oumin Shi, Qiang Liu, Haige Chen, Wei Xue, Yiran Huang

**Affiliations:** From the Department of Urology, Ren Ji Hospital, School of Medicine (LD, HC, WX, YH); School of Medicine (JH, LH); School of Public Health (OS); and Department of Pathology (QL), Ren Ji Hospital, School of Medicine, Shanghai Jiao Tong University, Shanghai, China.

## Abstract

Thyroid-like follicular carcinoma of the kidney (TLFCK) is an extremely rare subtype of renal cell carcinoma with close resemblance to the well-differentiated thyroid follicular neoplasms. TLFCK has not been included in the 2004 World Health Organization (WHO) classification due to the limited data available. Only 27 cases have been reported in the literature to date.

Herein, we report a unique case of TLFCK that presented as a striking skull and meningeal metastasis 5 years after the initial diagnosis; this is the first case of TLFCK with such a novel metastasis pattern. A 68-year-old woman was found to have a right renal lesion using computed tomography (CT) during her regular clinical follow-up visit for bladder cancer, but she exhibited no obvious clinical symptoms. The CT scan showed a 4.4-cm diameter, slightly lobulated soft tissue mass in the right lower kidney, the pathological findings of which showed a TLFCK. Five years later, the patient had progressed to skull and meningeal metastasis.

Both the renal tumor and the metastasis lesion were composed almost entirely of follicles with a dense, colloid-like material that resembled thyroid follicular carcinoma. However, no lesion was found in the thyroid gland. The neoplastic epithelial cells were strongly immunoreactive for cytokeratin 7 (and vimentin but negative for thyroid transcription factor-1 and thyroglobulin.

This is the first reported case of TLFCK to consist of widespread metastases to the skull and meninges and provides evidence that this rare variant of renal cell carcinoma has uncertain malignant potential and can be more clinically aggressive than previously believed.

## INTRODUCTION

Thyroid-like follicular carcinoma of the kidney (TLFCK) is an extremely rare subtype of renal cell carcinoma (RCC) that has low malignant potential and exhibits a striking histology that resembles well-differentiated thyroid follicular neoplasms.^1^ TLFCK, distinguished from kidney thyroidization and metastatic thyroid follicular carcinoma, is characterized as negative for thyroid immunohistochemical markers such as thyroid transcription factor-1 (TTF-1) and thyroglobulin (TG). TLFCK has not yet been included in the 2004 World Health Organization (WHO) classification,^1^ and the current consensus from the International Society of Urological Pathology (ISUP) is to not recommend TLFCK as a new WHO histological classification given the limited number of cases available for review.^2^ The first case was reported in 2004,^3^ and since then, an additional 26 cases have been reported in the literature.^5–26^

Herein, we report a unique case of TLFCK that presented as notable skull and meningeal metastases with a history of urothelium carcinoma of the bladder 6 years before the renal lesion was found. This is the first case of TLFCK to take the form of widespread metastases to the skull and meninges with a history of bladder neoplasm. In this article, we further discuss the clinical, histological, and immunohistochemical findings and provide a review of the available literature.

## CONSENT

Written informed consent was obtained from the patient for the publication of clinical data and images.

## CASE REPORT

In 2009, a 68-year-old woman underwent a computed tomography (CT) scan to explore a right renal occupancy during her regular clinical follow-up visit for bladder cancer, and she exhibited no obvious clinical symptoms. Before her retirement, she had worked in the chemical industry for over 10 years. Her past medical history included papillary urothelium carcinoma of the bladder (pTaN_0_M_0_, grade 2), which was diagnosed at age 62, with a clinical presentation of painless gross hematuria. The patient's bladder cancer was treated by transurethral resection of a bladder tumor (TURBT), and she was administered routine pirarubicin intravesical chemotherapy for 3 years. The bladder lesion recurred twice during the first 2 years and had remained undetectable until the time the renal lesion was identified. Her relevant family history included pancreatic cancer in her father.

**FIGURE 1:**
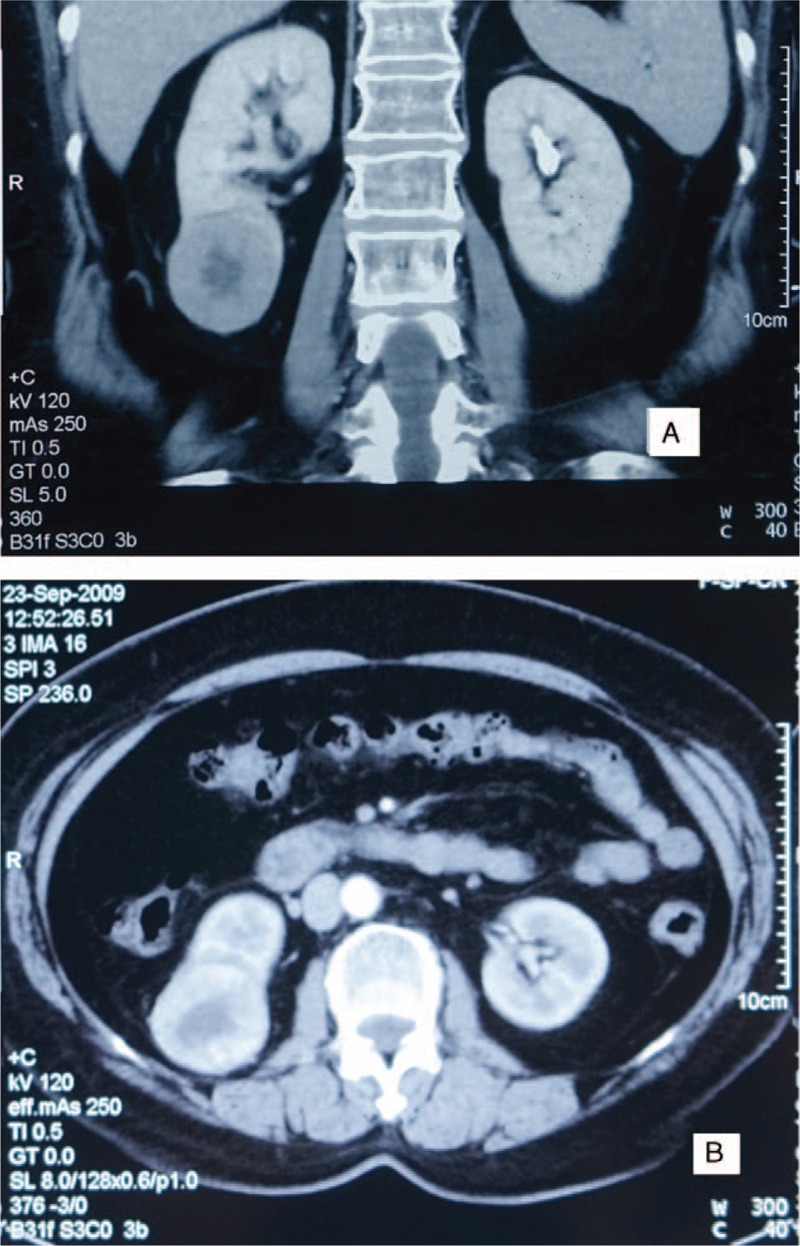
CT scans of TLFCK. (A) Coronal and (B) axial CT scans with contrast showing a 5 × 4.5 × 3-cm inhomogeneous and partially exophytic lesion in the right lower kidney, with hypodense, necrotic lesions in the middle surrounded by relatively hyperdense lesions. CT = computed tomography, TLFCK = thyroid-like follicular carcinoma of the kidney.

An abdominal CT scan showed a 5 × 4.5 × 3-cm inhomogeneous and partially exophytic lesion in the right lower kidney with hypodense, necrotic lesions in the middle surrounded by relatively hyperdense lesions (Figure 1). No metastatic lesions, lymph node enlargement or renal vein involvement was noted. After a radical laparoscopic nephrectomy, the pathological examination showed a 5 × 4.5 × 3-cm mass in the right lower kidney. Further immunohistochemical staining was performed (Table 1). The patient's thyroid gland was examined carefully after the surgery, and there were no significant pathologic findings. Her immediate postoperative course was uneventful, and at the 24-month postoperative follow-up visit, there was no evidence of tumor recurrence or metastatic disease.

**TABLE 1:**
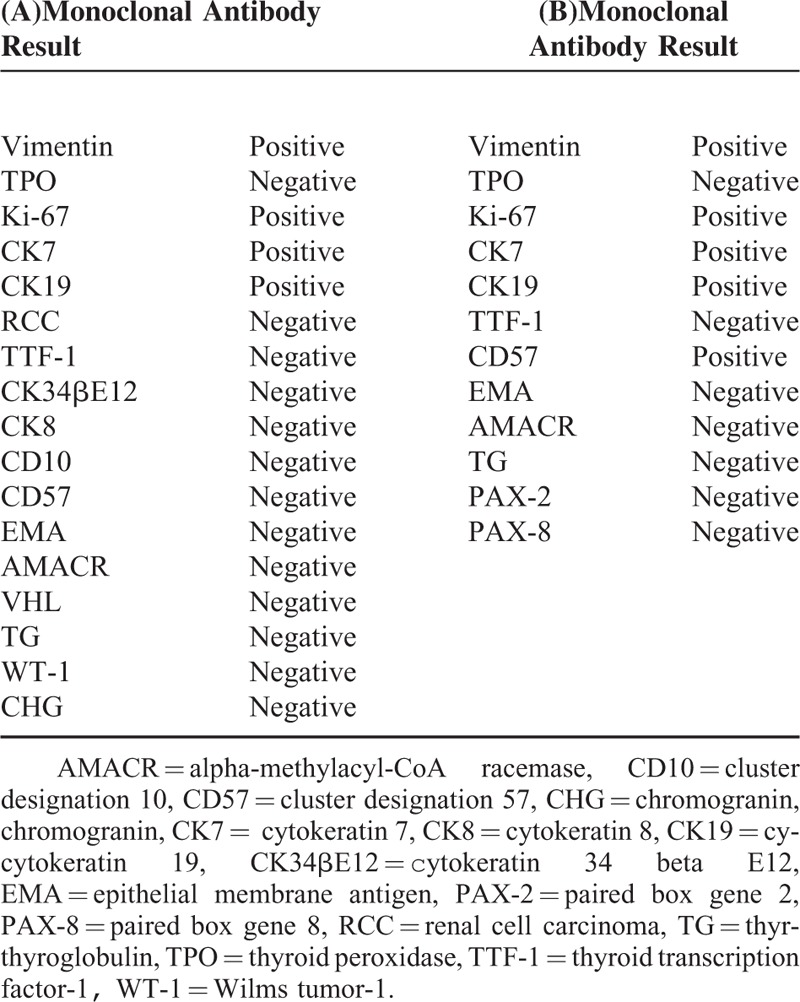
Antibodies Used for Immunohistochemical Staining and Results in Renal Primary Tumor (A) and Skull and Meningeal Metastasis (B)

The patient was then lost to follow-up until 2014, when she noticed there was a painless, progressively growing mass on the left top of her head; at that time, she was found to have skull and meningeal metastases in the left parietal area. The metastases were resected and diagnosed on histopathology as TLFCK, and cranioplasty was also performed. Pathological examination showed a soft, tan, bleeding mass (6 cm in diameter) in the left parietal area of the skull that presented bone destruction and a 4 × 3.5 × 0.8-cm irregular mass in one of the meninges. After a follow-up of 12 months, the patient remained asymptomatic.

## MATERIALS AND METHODS

**FIGURE 2:**
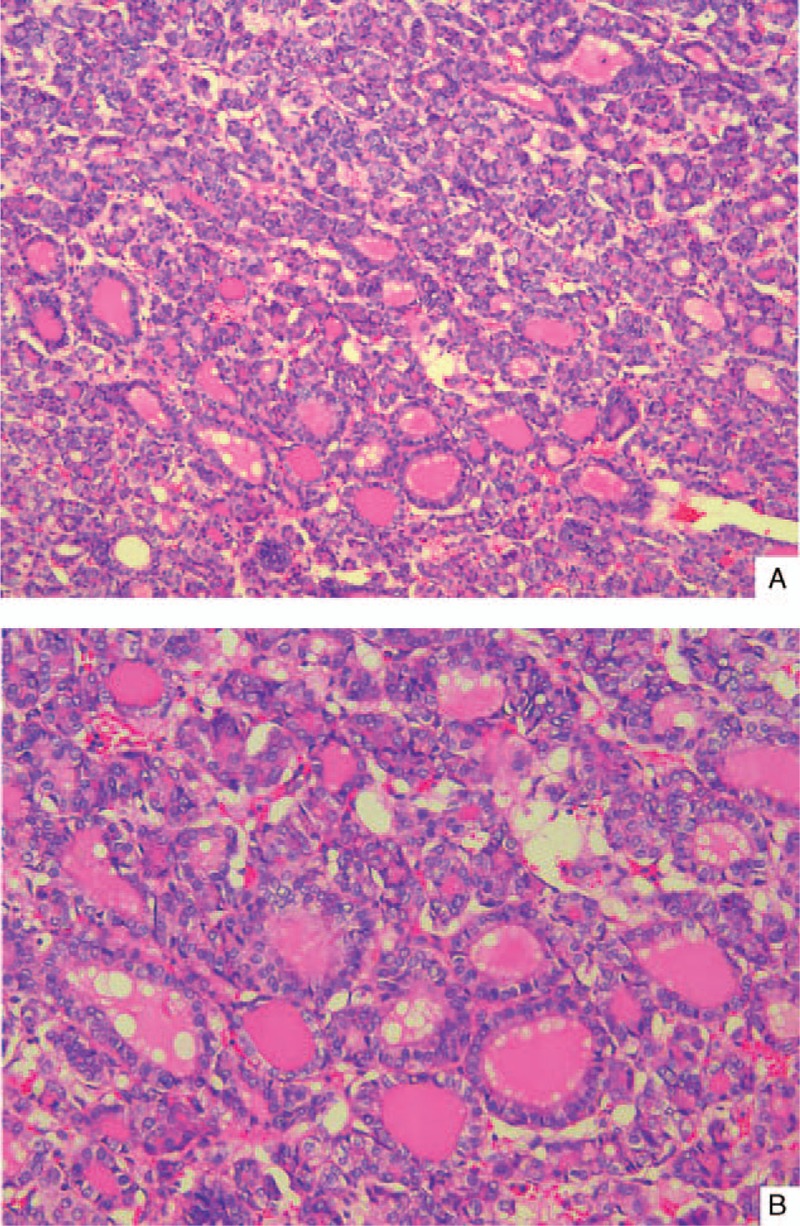
(A) The tumor demonstrated follicles of variably sized and shaped follicles filled with dense eosinophilic material under H&E staining (200 × objective). (B) In the microfollicular area, the nuclear features showed moderate anisonucleosis and were occasionally oval in shape under H&E staining (400 × objective). H&E = hematoxylin and eosin.

Both renal and metastatic surgical specimens were sampled according to the current protocols.^4^ Formalin-fixed, paraffin-embedded tissue samples were obtained, and 4-μm sections were stained with hematoxylin and eosin (H&E). The slides were fixed in 95% alcohol before H&E staining. The specimens were processed in 10% neutral-buffered formalin, and the H&E-stained sections were studied. Both primary and metastatic tumors histologically revealed epithelial follicular structures that consisted of macro- and microfollicles filled with eosinophilic amorphous colloid-like material reminiscent of follicular carcinoma of the thyroid gland (Figure 2). Mitoses were absent, while hemorrhagic areas and cholesterol crystals were present.

**FIGURE 3:**
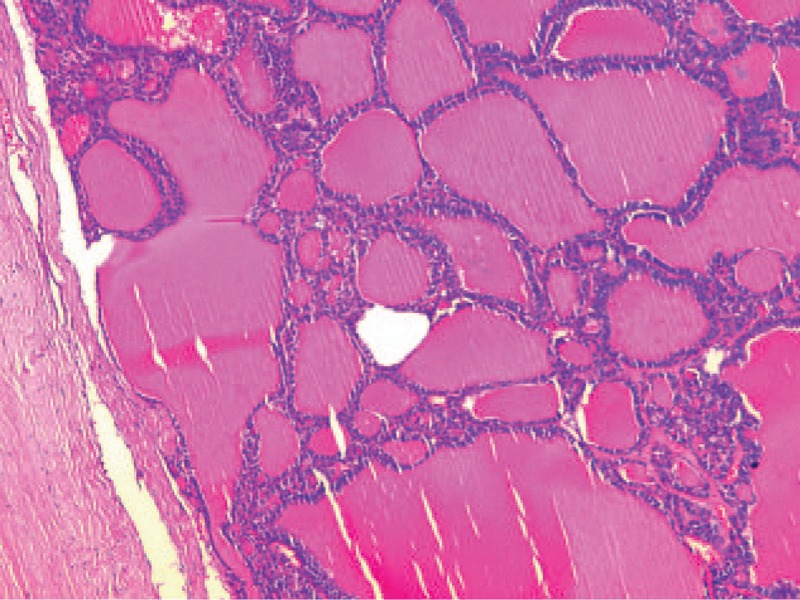
Metastatic TLFCK in the left parietal area of skull. The metastasis was composed of follicles of various sizes with colloid-like material and showed greater variability and complexity of follicle size and shape. TLFCK = thyroid-like follicular carcinoma of the kidney.

In the renal primary tumor, the tumor was unifocal and well circumscribed with a thin fibrous capsule. Some irregular macrofollicles and densely packed microfollicles filled with eosinophilic colloid-like material were present. All variably sized follicular architectures were formed by a single layer of cuboidal to columnar cells. Focally, solid areas with a pseudo-sarcomatoid appearance were observed, without well-formed follicles. The cells showed elongated cytoplasm with round-oval nuclei that varied slightly in size, and the cytoplasm and round nuclei contained evenly distributed chromatin with rare mitotic figures. No areas of typical conventional (clear cells) or other known types of RCCs were found. The tumor was limited by thin fibrotic stroma. Histologic examination of the relative lymph node revealed no metastasis. Surgical specimens of the left parietal area metastasis displayed a highly atypical parenchyma that consisted of follicles of varying size filled with larger amounts of eosinophilic colloid-like material compared with the renal lesion (Figure 3). The nuclei were round to oval with evenly distributed chromatin and inconspicuous nucleoli. Bone destruction was observed at this time.

**FIGURE 4:**
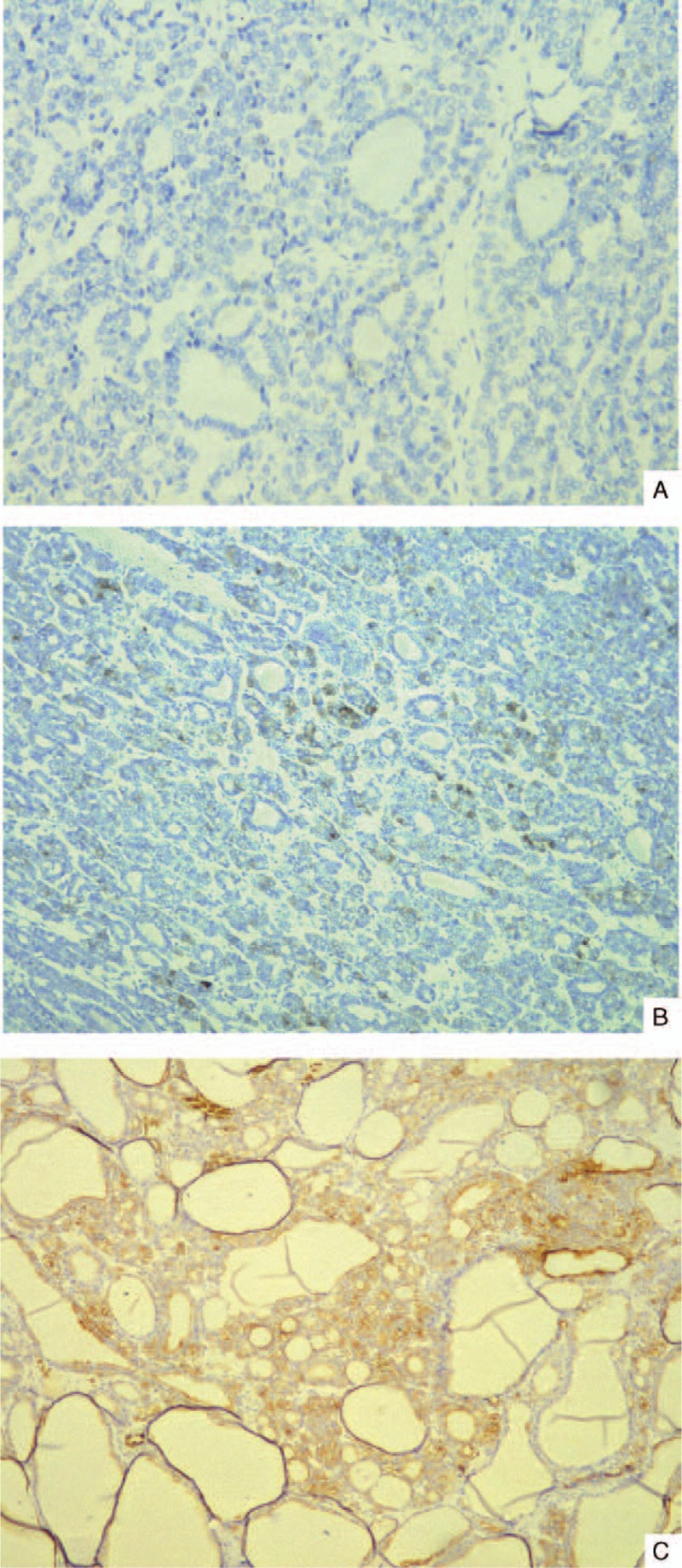
Histologic and immunophenotypic features of TLFCK. (A) Immunohistochemical staining of renal tumor cells showed no reactivity for TTF-1 (B) Immunohistochemical staining of renal tumor cells showed probable positive for TPO (C) Immunohistochemical staining of metastatic tumor cells showed reactivity for CK7. CK7 = cytokeratin 7, TLFCK = thyroid-like follicular carcinoma of the kidney, TPO = thyroid peroxidase, TTF-1 = thyroid transcription factor-1.

Immunohistochemistry was carried out using standard immunohistochemical techniques. The primary antibodies used were those for TTF-1, TG, thyroid peroxidase (TPO), pancytokeratin (AE1/AE3), cytokeratin 7 (CK7), epithelial membrane antigen (EMA), paired box gene 2 (PAX-2), paired box gene 8 (PAX-8), CD10, CD56, Wilms tumor-1 (WT-1), vimentin, and Ki-67. Sections were lightly counterstained with hematoxylin. Appropriate positive and negative controls were run concurrently for all of the applied antisera. In both lesions, immunohistochemical analysis showed identical results. The renal neoplastic epithelial cells were diffusely and strongly immunoreactive for CK7, vimentin, CK19, and Ki-67, and the tumor cells were negative for TTF-1 (Figure 4A), TG, RCC, CD57, CK8, CD177, alpha-methylacyl-CoA racemase (AMACR), VHL, CD10, 34βE12, WT-1, EMA, and chromogranin. The tumor cells were likely positive for TPO (Figure 4B). Immunohistochemical staining metastatic tumor cells revealed strong reactivity for vimentin, CK7 (Figure 4C), Ki-67/ck, and AE1/AE3. Other markers such as TTF-1, TG, and PAX-2 were negative (Table 1).

## RESULTS

Therefore, based on the morphology and immunohistochemical profile, a diagnosis of TLFCK in both the primary and metastatic tumors was confirmed. This patient's history and disease are remarkable. More importantly, this is the first case of TLFCK to present as widespread metastases to the skull and meninges after initial bladder carcinoma, providing evidence that this rare variant of renal cell carcinoma can be more clinically aggressive than previously believed.

## DISCUSSION

Here, we report a unique case of TLFCK in a 68-year-old woman with a history of papillary urothelium carcinoma of the bladder who presented with a distant skull and meningeal metastasis 5 years after initial diagnosis. TLFCK is a rare and recently described histologic variant of RCC that is not included in the current WHO classification of renal tumors.^1^ Since the first case was reported in 2004, only 26 cases of TLFCK have been reported in the literature.^5–26^ Although TLFCK shows histologic features that resemble thyroidal follicular carcinoma, the diagnosis of TLFCK can be confirmed through its morphology and immunohistochemical results. Physical examinations and radiographic studies have disclosed no thyroid lesions in any patient. Moreover, the tumor cells lack immunoreactivity for the thyroid-specific markers TG, TTF-1, and TPO.

## CLINICOPATHOLOGIC FEATURES

Clinicopathologic characteristics of previously reported cases and the present one are shown in Table 2, including 20 English-literature cases and 7 cases published in the Chinese and German literature. These tumors occur mainly in middle-aged adults (mean age: 43.0 years; range: 19–83 years), and over half of the affected patients were female (60.71%, 17/28). More than half of the patients with TLFCK were asymptomatic (15/28 cases, 53.57%), the same as in our case. In symptomatic patients, hematuria (8/28, 28.57%) and abdominal/flank pain (7/28, 25.00%) were the most common presentations; four patients complained of hematuria and abdominal/flank pain at the same time (4/28, 14.28%). Other symptoms such as weight loss^11^ and hypertension^19^ have also been reported. All previously described TLFCK cases have been solitary tumors, and the primary tumor sizes have ranged from 1.9 to 16.0 cm (mean: 5.0 cm). Nevertheless, no apparent correlation between tumor size and the development of metastatic disease was found according to the previous case reports (Table 2). The reported patients with TLFCK have survived, with a mean follow-up time of 20 months (range: 1–84 months); only one patient died of complications of acute myeloid leukemia after chemotherapy 18 days after hospitalization.^24^ Follow-up information was unavailable for 5 patients.^7,13,16,18,25^

**TABLE 2:**
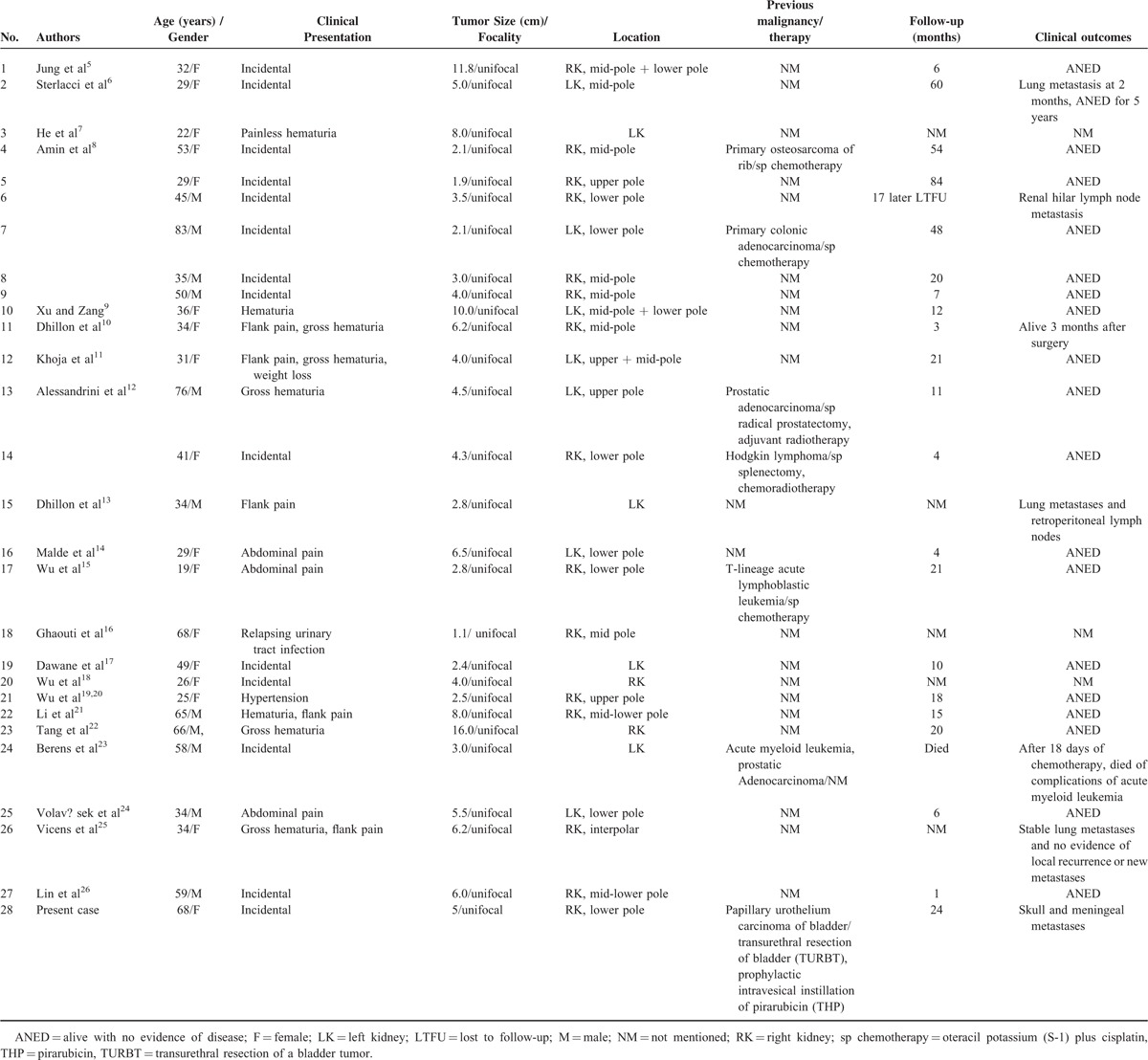
Clinicopathologic Characteristics of Previously Reported Cases and the Current Case

On gross examination, most tumors have been unifocal and surrounded by a thin fibrous capsule, with or without areas of cystic changes^12,14,24,26^ or necrosis.^5,12,25,26^ However, some cases showed nonencapsulated tumors.^11^ The tumor tissue was generally confined within the kidney parenchyma but rarely presented capsular invasion or extension into perinephric adipose tissue. On microscopic evaluation, the tumors have been composed of a follicular architecture lined by a single layer of cuboidal to columnar cells that form macro- and microfollicles that contain eosinophilic amorphous colloid-like material and resemble follicular carcinoma of the thyroid. The cuboidal-columnar cells show low-grade nuclei and hazy cell borders with round–oval nuclei and smoothly distributed chromatin. Most tumors are assigned Fuhrman nuclear grade 1 to 2 with occasional grade 3, and mitotic activity is absent or rare. The tumor in our case demonstrated features similar to previously reported TLFCK cases.

The immunohistochemical profiles of the previously reported TLFCK cases show that most tumors are positive for AE1/AE3 (100%, 10/10 cases), PAX-8 (100%, 4/4), CAM5.2 (100%, 3/3), CK19 (87.5%, 7/8), EMA (86.7%, 13/15), CK7 (80.0%, 20/25), and vimentin (70.8%, 17/24). Variable expression in TLFCK samples can be seen for CK34βE12 (50%, 3/6 cases), NSE (50%, 2/4), HBME-1 (50.0%, 2/4), PAX-2 (50.0%, 6/12), galectin-3 (33.3%, 1/3), CK20 (30.8%, 4/13), CD10 (30.4%, 7/23), CD99 (25%, 1/4), CD15 (16.7%, 1/6), AMACR (9.1%, 1/11), RCC markers (8.3%, 1/12), CD56 (7.1%, 1/14), and WT-1 (6.2%, 1/16). TLFCK samples are negative for TTF-1 (100%, 0/26 cases), TG (100%, 0/26), CD57 (100%, 0/10), synaptophysin (100%, 0/8), CD117 (100%, 0/7), CEA (100%, 0/5), and TPO (100%, 0/1). The most important pathological feature of the reported cases of TLFCK is consistent immunonegativity for the thyroid-specific markers TTF-1 and TG. Our patient's tumor cells were negative for TTF-1 and TG in the renal and skull and meningeal tissues. Although the TPO immunostain for this patient's tumor cells was positive in a few single cells, the vast majority of cells showed negative staining. We repeated the immunohistochemical staining for TPO in 2015 and obtained the same outcomes.

### Differential Diagnosis

It is essential to distinguish TLFCK from renal metastases of thyroid carcinoma and other primary renal tumors with thyroid-like features, as well as from thyroidization of renal tubules. The differential diagnosis of TLFCK is necessary because the treatments for TLFCK and mirrored diseases are quite different.

Although they are very rare, metastases to the kidney from the thyroid have been reported.^27^ To our knowledge, 21 cases have been reported in the English-language literature and another 30 in the Japanese literature.^27^ Of the 21 cases of renal metastasis associated with differentiated thyroid carcinoma (DTC), 9 were from papillary carcinoma (6 were follicular-variant papillary carcinomas) and 12 were from follicular carcinoma.^28^ TLFCK usually presents as a solitary tumor, but the majority of thyroid carcinoma metastases to the kidney are widely disseminated. More importantly, metastatic thyroid carcinoma is immunoreactive for TTF-1 and TG, but TLFCK is not. Treatment of thyroid metastases to the kidney involves total thyroidectomy, resection of the metastatic foci, and 131I therapy to remove any residual disease or micrometastases.^29^

Thyroid-like features can also occur in other primary renal tumors. For instance, these features have been documented in papillary RCC.^30,31^ Meanwhile, the thyroid-like appearance in other subtypes of RCC is rare and usually focal. Conversely, TLFCK was unifocal in the presented cases, showing entirely follicular morphology filled with colloid-like material.^16^ Moreover, between TLFCK and common subtypes of RCC including clear cell carcinoma, metanephric adenoma, and oncocytoma, the tubules of papillary RCC differ greatly. For instance, in thyroid-like papillary RCC, the tumors still contain the classic papillary carcinoma structure, although this may be combined with some thyroid-like areas according to previous reports. TLFCK tumors, however, are composed entirely of thyroid-like macro- and microfollicles that contain eosinophilic colloid-like material, as outlined previously. Additionally, thyroid-like follicle spaces that contain eosinophilic material can also be found in tumors such as oncocytoma^32^ and metanephric adenoma,^31^ but their histological features are quite different from TLFCK.

Thyroidization occurs as a process that is secondary to chronic pyelonephritis or obstructive uropathy, and it is a common characteristic of end-stage renal diseases.^33^ TLFCK presents as a well-circumscribed mass surrounded by a thin fibrous capsule; in contrast, thyroidization of renal tubules often shows a typically widespread mass in the bilateral kidney. Furthermore, TLFCK usually occurs in patients without renal disease. However, a recent case reported a patient with TLFCK who had a history of concomitant autosomal dominant polycystic kidney disease (ADPKD).^26^

More data on genetic alterations are needed to safely draw conclusions and clarify differential diagnoses in cases of TLFCK, given the various genetic abnormalities that have been recognized. Using comparative genomic hybridization analysis, Jung et al^5^ reported losses of chromosomes 1p36, 3 and 9q21–33 and gains of chromosomes 7q36, 8q24, 12, 16, 17p11-q11, 17q24, 19q, 20q13, 21q22.3, and Xp. Sterlacci et al^6^ identified losses of chromosomes 1, 3, 7, 9p21, 12, 17, and X in this type of tumor using fluorescent in situ hybridization (FISH) analysis. Wu et al^15^ failed to demonstrate translocations or amplifications of the MLL gene, although Dawane et al^17^ found borderline monosomies in both chromosomes 7 and 17 by FISH analysis.

### Metastasis and Invasiveness

Our case is the first to report bone and meningeal metastasis. To date, TLFCK followed by metastatic disease has been observed in five patients,^6,8,10,25^ including the patient described herein. These previous cases include a 29-year-old female patient who developed a lung metastasis 2 months after initial diagnosis,^6^ a 45-year-old male patient who presented with a renal hilar lymph node metastasis,^8^ a 34-year-old male patient who was found to have lymph node and lung metastases at the time of presentation,^10^ and one 34-year-old female patient who presented with lung and lymph node metastases at initial diagnosis with stable lung metastases and no evidence of local recurrence or new metastases.^25^ Our patient presented with a striking skull and meningeal metastasis 3 years after the initial diagnosis, which is quite different from the previous cases, indicating a new presentation of distant TLFCK metastasis. Based on the types of metastasis reported to date, the most likely metastasis route of TLFCK is hematogenous, as previous therapy did not fully eliminate TLFCK cells, which subsequently recurred and metastasized.

TLFCK is regarded as possessing medium invasiveness.^10,26^ However, as seen in the prior four case reports,^6,8,10,25^ this tumor can be quite invasive. The tumor cells in our case likely showed a higher level of malignancy than the other cases; although Dawane et al^17^ furnished evidence of a relatively slow-growing increase in tumor size (5 years only witnessed a slight increase from 2 to 2.5 cm), the skull and meningeal metastases in our case had emerged and enlarged to 4 cm within 3 years. Long-term survival could be achieved by radical resection of the tumor in most of the previous cases. For some metastatic cases, a radical nephrectomy with additional extensive lymph node dissection was performed.^6^

### Past Medical History of Other Malignancies and Chemotherapy

Previous malignancies for TLFCK include osteosarcoma of the rib,^8^ adenocarcinomas of the colon^8^ and prostate,^12^ Hodgkin lymphoma,^12^ acute myeloid leukemia and prostatic adenocarcinoma,^23^ T-lineage acute lymphoblastic leukemia (T-ALL),^15^ and papillary urothelium carcinoma of the bladder in our case. Our patient was diagnosed with bladder carcinoma and received intravesical chemotherapy with pirarubicin (THP) for 3 years after her first TURBT. Due to the limited number of cases reported, there is no evidence to suggest any association between TLFCK and other malignancies or urinary diseases. One case of TLFCK was reported in a patient with ADPKD.^26^ The association between acquired polycystic kidney disease and renal tumors has been well documented, and this type of tumor has been included in the WHO classification. However, the association between ADPKD and TLFCK remains unclear. TLFCK patients with history of a previous malignancy (25.00%, 7/28) (Table 1) were slightly older (mean age: 56.8 years; 19–83 years) than other patients (mean age 39.6 years; 22–68 years). The youngest patient with T-ALL was 19 years old, which represents the only case of a patient who was younger than middle age; excluding this patient as an outlier, the mean age of TLFCK patients is 63.2 years. Hence, there is the potential for different pathogenic mechanisms between young and old TLFCK patients.

Additionally, chemotherapeutic regimens used to treat previous malignancies may predispose a patient to developing TLFCK. Indeed, a recent report concluded that receiving platinum-based chemotherapy increased the risk of kidney cancer as a second malignancy by 3.5 (95% CI: 1.0–11.2).^34^ Surprisingly, the majority of patients who suffered TLFCK as a second cancer received the oteracil potassium (S-1) plus cisplatin (SP) regimen for chemotherapy in the treatment of previous carcinomas.^8,12,15^ The most recent study identified a significantly increased risk of a second kidney carcinoma associated with the use of platinum-based agents as well as newly introduced chemotherapy agents.^35^ However, that study failed to identify any specific drug associated with the effect.^35^ Due to the limited number of cases reported, the connection between previous malignancies, chemotherapeutic treatment, and the development of TLFCK remains to be determined in future research.

## CONCLUSION

In summary, TLFCK is an extremely rare variant of RCC. In recent reports, TLFCK has shown uncertain malignant potential. Herein, we describe an unusual case of TLFCK that was the first to present with skull and meningeal metastasis. Our case provides further evidence of distant metastases in TLFCK, with a particularly aggressive behavior, and suggests that these tumors likely metastasize via the hematogenous route. Due to the limited number of cases reported, the ISUP does not intend to recommend TLFCK as a new WHO histologic classification. However, it is important to recognize this type of tumor and note the potential for metastasis to avoid inappropriate treatment due to misdiagnosis.
